# Novel coronavirus disease (COVID-19) in children

**DOI:** 10.3906/sag-2004-174

**Published:** 2020-04-21

**Authors:** Hasan TEZER, Tuğba BEDİR DEMİRDAĞ

**Affiliations:** 1 Department of Pediatric Infectious Disease, Faculty of Medicine, Gazi University, Ankara Turkey; 2 Department of Pediatric Infectious Disease, Ankara City Hospital, Ankara Turkey

**Keywords:** Novel corona virus, COVID-19, pediatrics

## Abstract

Coronavirus disease (COVID-19) was firstly reported at the end of 2019. The disease rapidly spread all around the world in a few months and was declared a worldwide pandemic by WHO in March 2020. By April 9, there were 1,436,198 confirmed COVID-19 cases in the world, nearly with 6% mortality rate. This novel infectious disease causes respiratory tract illness that may generally occur as mild upper respiratory tract disease or pneumonia. In older patients and/or patients with underlying conditions, it may result in acute respiratory distress syndrome, multi organ failure and even death. According to the current literature, children account approximately for 1%–5% of diagnosed COVID-19 cases. Generally, COVID-19 seems to be a less severe disease for children than adults. Approximately 90% of pediatric patients are diagnosed as asymptomatic, mild, or moderate disease. However, up to 6.7% of cases may be severe. Severe illness is generally seen in patients smaller than 1 year of age and patients who have underlying disesases. The epidemiological and clinical patterns of COVID-19 and treatment approaches in pediatric patients still remain unclear although many pediatric reports are published. This review aims to summarize the current epidemics, clinical presentations, diagnosis, and treatment of COVID-19 in pediatric patients.

## 1. Introduction

Many cases of pneumonia with an unknown origin were observed in Wuhan, Hubei Province, China [1,2]. It was reported that most of these patients exposed to the Huanan Seafood Wholesale Market. The disease spread rapidly, to other parts of China, and then globally, to many countries across six continents. 

On January 3, 2020, the Chinese Center for Disease Control and Prevention (China CDC) confirmed a novel member of enveloped RNA coronavirus as the cause of this disease^1^1Tan WJ, Zhao X, Ma XJ. A novel coronavirus genome identified in a cluster of pneumonia cases-Wuhan, China 2019−2020. China CDC Weekly 2020; 2:61-2.. The World Health Organization (WHO) described it as the 2019 novel coronavirus (2019-nCoV) on January 7, 2020. After a short period, WHO has declared the COVID-19 a public health emergency of international concern on January 30^2^2World Health Organization (2020). WHO Director-General’s statement on IHR Emergency Committee on Novel Coronavirus (2019- nCoV) [online]. Website https://www.who.int/dg/speeches/detail/who-director-general-s-statement-on-ihr-emergency-committeeon- novel-coronavirus-(2019-nCov) [accessed 03 March 2020].. Since then, the disease affected more than 177 countries globally. On February 11, 2020, this illness was named as the 2019 coronavirus disease (COVID-19) by WHO [3]. 

The first cases detected in Europe were reported from France on January 24, 2020, many European countries reported cases following France. In a short time, many people from many different countries in Europe were affected [4]. The first case from Turkey was reported on March 13, 2020. By April 9, there were 1,436,198 confirmed COVID-19 cases in the world, and total deaths were 85,522^3^3World Health Organization (2020). Coronavirus disease 2019 (COVID-19) Situation Report – 80 [online]. Website https://www.who.int/docs/default-source/coronaviruse/situation-reports/20200409-sitrep-80-covid-19.pdf?sfvrsn=1b685d64_4 [accessed 10 April 2020]..

The first confirmed pediatric case of Severe Acute Respiratory Syndrome (SARS)-CoV-2 infection was reported in Shenzhen on January 20 [5], and by January 31, more than 20 pediatric cases were reported in China [6]. Thereafter, many pediatric case reports and case series were reported. But the epidemiological and clinical patterns of the COVID-19 in pediatric patients still remain largely unclear despite the worldwide spread [2]. This report aims to identify the epidemiological characteristics, clinical findings, and treatment suggestions in pediatric patients with the 2019 novel coronavirus disease. 

## 2. Virology and pathogenesis

Coronaviruses (CoVs) are a group of related zoonotic viruses that cause disease in mammals and birds. They are enveloped positive-stranded RNA viruses with a crown-like appearance under an electron microscope, because of the spike glycoproteins on the envelope [7]. 

Coronaviridea family constitute the subfamily Orthocoronavirinae. Orthocoronavirinae subfamily classifies into four genera of CoVs: Alphacoronavirus (alphaCoV), Betacoronavirus (betaCoV), Deltacoronavirus (deltaCoV), and Gammacoronavirus (gammaCoV). Alpha-corona viruses include species of Human coronavirus 229E, Human coronavirus NL63, which concern human illnesses. Beta-corona viruses genus divides into four lineages (subgroup A, B, C and D) [8]. Subgroup A includes Betacoronavirus 1 (Human coronavirus OC43) and Human coronavirus HKU1 as human pathogens. Subgroup B includes Severe acute respiratory syndrome-related coronavirus (SARS-CoV, SARS-CoV-2). Subgroup C includes the Middle East respiratory syndrome-related coronavirus as a human pathogen [8]. 

Common human coronaviruses are; HCoV-OC43, HCoV-HKU1 HCoV-229E, and HCoV-NL63. They generally cause common cold and mild upper respiratory infections in immunocompetent individuals. Lower respiratory infections may occur in older or immune-compromised people [8]. 

Other important human CoVs are; SARS-CoV, SARS-CoV-2, and Middle East Respiratory Syndrome (MERS)-CoV. They cause epidemics with variable clinical severity presenting with respiratory and extra-respiratory manifestations. Concerning SARS-CoV, MERS-CoV, the mortality rates are up to 10% and 35%, respectively. As SARS-CoV-2 belongs to the beta-CoVs category, it has round or elliptic and often pleomorphic form, and a diameter of approximately 60–140 nm [8]. 

When it was evaluated genetically, the consistency of whole genome-wide nucleotide sequences of 2019-nCoV was consistent with SARS-like coronavirus in bats (bat-SL-CoVZC45) and the accordance ranged from 86.9% [9] to 89% [5], and 82% with that of human SARS-CoV [10]. 

According to this finding, the new virus was called SARS-CoV-2. The single-stranded RNA genome of the virus contains 29891 nucleotides, encoding for 9860 amino acids. These genomic analyses suggest that SARS-CoV-2 probably evolved from a strain found in bats, but its origins are not entirely understood [8].

SARS-CoV-2 is known to be sensitive to ultraviolet rays and heat. Like other coronaviruses, these viruses can be inactivated by lipid solvents, including either (75%), ethanol, chlorine-containing disinfectant, peroxyacetic acid and chloroform except for chlorhexidine [6,8].

The structure of the receptor-binding gene region is very similar to that of the SARS coronavirus, and the virus has been shown to use the same receptor, the angiotensin-converting enzyme 2 (ACE2), for cell entry [9,11]. When the virus enters the cell, the viral genome begins to replicate and translates structural proteins. After the process inside the cytoplasm, the vesicles containing the virus particles fuse with the plasma membrane to release the virus. After the entry of virus to the cell, antigen presentation cells (APC) -the main part of antiviral immunity- begin to present the antigens. Afterward, antigen presentation subsequently stimulates virus-specific B and T cells and they mediate the body’s humoral immunity and immunoglobulin M and G production begins. Specific IgM antibodies disappear approximately at the end of 12th week. IgG antibody lasts for a longer time and the SARS-specific IgG antibodies primarily are S-specific and N-specific antibodies [12]. There are more researches on the cellular immunity of coronavirus compared to humoral immunity [8]. The current data seems to indicate that the viral infection is capable of producing an excessive immune reaction in the host. In some cases, a ‘cytokine storm’ occurs of which the effect is extensive tissue damage. Interleukin 6 (IL-6) triggers this storm, which is produced by activated leukocytes and acts on a large number of cells and tissues. This cytokine storm may result in an acute systemic inflammatory syndrome characterized by fever and multiple organ dysfunction [8].

A report in Lancet showed that acute respiratory distress syndrome (ARDS) is the main cause of death in COVID-19 patients. ARDS is known as the common immunopathological result for SARS-CoV-2, SARS-CoV and MERS-CoV infections. The cytokine storm results with a violent attack by the immune system to the body, which cause ARDS and multiple organ failure, and finally lead to death in severe cases of SARS-CoV-2 infection, just like in SARS-CoV and MERS-CoV infection [13]. The cytokine storm and the deadly uncontrolled systemic inflammatory response resulting from the release of large amounts of proinflammatory cytokines (IFN-a, IFN-g, IL-1b, IL-6, IL-12, IL-18, IL-33, TNF-a, TGFb, etc.) and chemokines (CCL2, CCL3, CCL5, CXCL8, CXCL9, CXCL10, etc.) by immune effector cells ends with ARDS in SARS-CoV infection [14]. Similar to those with SARS-CoV, individuals with severe MERS-CoV infection show elevated levels of IL-6, IFN-a, and CCL5, CXCL8, CXCL-10 in serum compared to those with the mild-moderate disease [11]. 

It is noted that, COVID-19 children are less susceptible to COVID-19 and that seem to be affected less commonly than adults [3,15]. Suggested reasons include having a more active innate immune response, healthier respiratory tracts (because of not exposing to cigarette smoke and air pollution as adults), and fewer underlying disorders. In addition, they are generally overprotected by parents, and if there is not any positive case in family, the possibility of being infected decreases [15]. A dangerous and uncontrolled immune response in adults may also explain with a robust immune response which may lead to ARDS [15]. It was found that ACE2 expression in rat lung has been found to dramatically decrease with age [15]. This finding may not be consistent with a relatively low susceptibility of children to COVID-19. On the other hand, studies show that ACE2 is involved in protective mechanisms of the lung. It may protect against severe lung injury induced by respiratory virus infection in an experimental mouse model and in pediatric patients [16,17]. The difference between adults and children in case of severity may be related to differences in receptors in the Renin-angiotensin system (RAS) and altered inflammatory responses to pathogens [18].

## 3. Transmission

The first cases of the COVID-19 had the history of direct exposure to the Huanan Seafood Wholesale Market of Wuhan. So the animal-to-human transmission was presumed as the main mechanism at the beginning. In a short period, subsequent cases without any history of visiting the market were reported. Hence, it was stated that the virus could also be transmitted from human-to-human. The most frequent source of COVID-19 spread are symptomatic patients [8]. It is certain that the main sources of the infection are patients infected by 2019-nCoV with or without clinical symptoms [5,6], but in addition, patients in the incubation period may also have potency to transmit the virus. It is suggested that individuals who remain asymptomatic during disease could transmit the virus too [8].

According to the current data, the novel virus is primarily transmitted through respiratory droplets and contact routes4 World Health Organization (2020). Modes of transmission of virus causing COVID-19: implications for IPC precaution recommendations. Scientific brief. 29 March 2020 [online]. Website u29c1 [accessed 10 April 2020].. Respiratory droplets transmit the virus when patients cough, talk loudly or sneeze and transmission is possible via close contact (e.g., contact with the mouth, nose or eye conjunctiva by a contaminated hand) [6].

According to the data reported by the China Center for Disease Control (CDC) and local CDCs, the incubation time is generally 3 to 7 days and up to 2 weeks as the longest time from infection to symptoms was 12.5 days (95% CI, 9.2–18) [2]. On average, each patient transmits the infection to an additional 2.2 individuals. Remarkably, estimations of the R0 of the SARS-CoV epidemic in 2002–2003 were approximately 3 [19].

No airborne transmission was reported in an analysis of 75,465 COVID-19 cases in China [20]. Airborne transmission may be possible in only specific situations and settings in which procedures or support treatments that generate aerosols are performed, this transmission route especially concerns health care personnel.^4^4World Health Organization (2020). Modes of transmission of virus causing COVID-19: implications for IPC precaution recommendations. Scientific brief. 29 March 2020 [online]. Website https://www.who.int/news-room/commentaries/detail/modes-oftransmission- of-virus-causing-covid-19-implications-for-ipc-precaution-recommendations [accessed 10 April 2020].

There is some evidence that COVID-19 infection may result with intestinal infection and virus can be present in faeces. However, there have been no reports of faecal-oral transmission of the COVID-19 virus to date.4 In a study of Xing et al., clearance of SARS-CoV-2 in respiratory tract occurred within two weeks after the decrease in fever, whereas viral RNA remained detectable in stools of pediatric patients for longer than 4 weeks [21]. 

Mother to infant transmission of SARS-CoV-2 is controversial (through breast-milk or vertical transmission). In pregnancy, viremia rates seem to be low, shown as 1% in Wang’s report [22]. This finding suggests that placental seeding and vertical transmission is unlikely. In Schwartz’s review, 38 pregnant women with COVID-19 were reported, and, no cases of intrauterine transmission were documented [23]. On the other hand, many possible cases have been reported that presenting newborns delivered from COVID-19 pregnants, with increased Ig m levels but negative polymerase chain reaction tests results after cesarean delivery or reports presenting neonatal pneumonia due to COVID-19, with nasopharyngeal and anal cultures and polymerase chain reaction tests were positive for SARS-CoV-2. 

But positive IgM results alone are not definitive evidence of in utero infection. So early infant infection may have been due to postnatal contact with infected parents or caregivers [24]. Thus, there is a suspect, but no certain evidence of vertical transmission for COVID-19 yet and further reports and studies are warranted. 

Transmission with breast milk is another matter of debate. It is not clear if the virus transmits through breast milk or via feeding the baby. According to Wang’s report, it is suggested that infants should not be fed with breast milk from mothers with neither confirmed nor suspected of 2019-nCoV. If breast milk of these mothers tested negative for 2019-nCoV, then infants may be fed with breast milk. Using donor milk can be considered after being screened for 2019-nCoV, in case of excretion of the virus into the milk during the incubation period [25]. On contrary, another study presented that no virus was found in the maternal milk of six COVID-19 patients [26]. Another recent study by Davanzo et al, according to the latest data, it was advised that if a mother diagnosed or suspected as COVID‐19 or under investigation for COVID‐19 whether asymptomatic or paucisymptomatic at delivery, breastfeeding is advisable, under strict measures of infection control [27].

In concordance with this study when a mother with COVID‐19 is too sick to care for the newborn, the neonate will be managed separately and fed fresh expressed breast milk, with no need to pasteurize it, as human milk is not believed to be a vehicle of COVID‐19 [27]. WHO suggests that women with COVID-19 can breastfeed if they wish to do so. However, it is mentioned that there is still a risk for droplet transmission via close contact during feeding (breastfeeding or bottle feeding). So, it is important to carry out precautions to prevent transmission. Mothers should practice respiratory hygiene during feeding, wearing a mask where available, wash hands before and after touching the baby, and routinely clean and disinfect what they have touched^5^5World Health Organization (2020). Q&A on COVID-19, pregnancy, childbirth and breastfeeding [online]. Website https://www.who. int/news-room/q-a-detail/q-a-on-covid-19-pregnancy-childbirth-and-breastfeeding [accessed 10 April 2020].. 

## 4. Epidemiology

Pediatric cases reported during the previous outbreaks of SARS in Hong Kong and MERS in South Korean, were very few [28]. In addition, mortality rate of SARS and MERS in the adults was very high, but there were no fatalities in the pediatric cases [28]. These findings indicated that children appeared to have a milder form of the disease caused by the coronaviruses [2]. Current data about COVID-19, in pediatrics is similar to SARS and MERS in case of disease severity and mortality and shows that children of all ages can get COVID-19, but they seem to be affected less commonly than adults [3]^6^6Centers for Disease Control and Prevention (2020). Coronavirus Disease 2019 in Children–United States, February 12–April 2, 2020 [online]. Website https://www.cdc.gov/mmwr/volumes/69/wr/mm6914e4.htm [accessed 10 April 2020]..

The first confirmed pediatric case of SARS-CoV-2 infection was reported in Shenzhen on January 20 [5], and by February 10, a total of 398 confirmed pediatric cases were reported from China, excluding the Hubei Province [2]. It is well known that pediatric cases are generally identified at that time in a familial cluster or and generally are infected by one sick parent or family member. According to a study, 71.2% (183/257) of infected children were reported having a household contact [29]. In another study by Lu, 90.1% of patients were reported to be with family clusters [30]. But at the explosion stage of the outbreak, children may become a significant spreader [31].

The surveillance definitions and criteria are changing during the pandemic, but still data from many different countries, are similar with these studies and the proportion of pediatric cases is within this range. 

Less than 1% of the cases were in children younger than 10 years of age in the review of 72,314 cases reported by the Chinese Center for Disease Control and Prevention [32]. According to a study that analyzed 44,672 COVID-19 cases from China by mid-February 2020, 0.9% of patients were less than 10 years of age and 1.2% were between 10 and 20 years of age [33]. According to Statista Research Department, Italy, 1.6% of total patients were from 0 to18 age^7^7Statista (2020). Distribution of coronavirus cases in Italy, as of April 15, 2020 by age group [online]. Website u29c8 [accessed 10 April 2020].. In a systematic literature review (between January 1and March 18, 2020), children were 1 to 5 percent of diagnosed COVID-19 cases [34]. To date, there are about 149,760 laboratory-confirmed cases reported from United States, and only 1.7 percent are in children.^6^

In a report of 171 pediatric cases, reported by Lu et al., median age of patients was 6,7 year (1 day–15 years) and 60.8% were male.

 In this case series, 18.1% of patients were <1 year of age, 23.4% were 1–5 years, 33.9% were 6–10 years, 24.6% were 11–15 years [30]. In Dong’s study, there were 728 confirmed cases and 1407 suspected cases. 17.6% of pediatric cases were <1 year of age, %23 were 1–5 years, 24.5% were 6–10 years, %19.3 were 11–15 years, %15.6 were >15 years. The median age of all patients was 7 years and 56.6% of patients were male [3]. According to US data, among all 2,572 COVID-19 cases in children, the median age was 11 years (0–17 years). Approximately one-third of reported pediatric cases 32% were between 15–17 years of age, 27% were between 10-14 years of age. Fifteen percent of cases were aged <1 year, 11% of cases were aged 1–4 years, and 15% of cases were aged 5–9 years. Males comprised 57% of cases in the population in which sex information was obtained; among 184 cases in children whose information of exposure were available, 9 % had a history of travel, and 91% had a history of contact with a COVID-19 patient in the household or community.^6^

By 30.03.2020, there were 11535 total cases, of which 117 (1%) were pediatric in Turkey. The mean age was 8 years (1day–17 years). The 13.6% of cases were <1 year. There were 3 neonatal cases. Nearly 53% of cases were male. Patients with a history of contact were 48.7%. Only 1.7% of cases had a history of travel abroad. Turkish citizens comprised 93.1% of cases, but 6.9% were immigrants^8^8Turkish Republic Ministry of Health (2020), COVID19 Surveillance data (unpublished)..

## 5. Clinical findings

According to the available data, COVID-19 in children appears to be usually mild. A minority of children with COVID-19 require hospitalization. By March 6, among 2572 laboratory-confirmed cases of COVID-19 in children reported from the US, the estimated rate of hospitalization differed from 6% to 20%, and 0.58%–2.0% of them were admitted to an ICU.6 In this report, children aged <1 year had the highest percentage (15%–62%) of hospitalization among pediatric patients with COVID-19. Among 95 children aged <1 year with known hospitalization status, 59 (62%) were hospitalized, including five who were admitted to an ICU. The percentage of patients hospitalized among those aged 1–17 years was lower (estimated range = 4.1%–14%), with little variation among age groups.^6^

Fever and cough are the most common reported symptoms in children [30]. Fever (subjective or documented), cough, and shortness of breath were more common among adult patients aged 18–64 years (93% reported at least one of these). 

In contrast, these signs and symptoms were less frequently reported among pediatric patients (73%). In the case series from the United States, complete information about symptoms was available for 291 children; 56 percent had a fever, 54 percent had a cough, and 13 percent has shortness of breath; at least one symptom was observed in 73% of children6 On the other hand, these ratios were 71%, 80%, and 43%, respectively, in adults. Sore throat, myalgia, headache, and diarrhea were also reported rarely by pediatric patients.^6^

In another series of 1391 children evaluated for COVID-19 at Wuhan Children’s Hospital, 171 (12%) had confirmed SARS-CoV-2 infection (by identification of RNA). In this report, 15.8% percent of children with confirmed infection were asymptomatic, 19.3% had upper respiratory infection, and 64.9% had pneumonia. Fever was the most common symptom, occurring at some point in illness in approximately 41.5%. Other common symptoms included cough (48.5 percent) and pharyngeal erythema (46.2%). Less common symptoms included fatigue, rhinorrhea/nasal congestion, diarrhea, and vomiting. (changing between 5% and 9%). A total of 12 asymptomatic patients had radiologic features of pneumonia. A total of 21 patients were in stable condition in the general wards, and 149 were discharged from the hospital [30].

According to Dong et al.’s report, regarding the severity, 94 (4.4%), 1088 (51.0%), and 826 (38.7%) cases were diagnosed as asymptomatic, mild, or moderate, respectively; and accounted for 94.1% of all cases. Severe and critical cases were 6.7% and 0.7% of patients [3]. The clinical features, laboratory testing, and chest radiograph imaging state the severity of COVID-19 [35]. Similar clinical manifestations have been reported in smaller case series from China [36–38]. 

According to the surveillance data from Turkey, 50.4% of pediatric cases had a mild disease, and 0.8% had severe disease. Intensive care hospitalization rate was 4.27% and 80% of them were under one year of age.^8^

Another critical age group in pediatrics is the neonatal period. Neonates are generally associated with milder disease [26,39]. In a report about COVID-19 in the neonatal period, it was found that the clinical symptoms of 33 neonates with or at risk of COVID-19 were obscure, and favorable outcomes were seen. 

Of 33 neonates born to COVID 19 mothers, 30 had negative test results, but there were 3 neonates with symptomatic COVID-19. The most seriously ill neonate was possibly symptomatic because of prematurity, asphyxia, and sepsis, rather than SARS-CoV-2 infection [40]. In a case series of neonates, born to COVID-19 mothers, the 2019-nCoV nucleic amplification test results were negative for all neonates; thus there was no evidence of vertical transmission of 2019- nCoV via the placenta and they were all fine, so no antiviral treatment was administered to the neonates [41].

Although most children appear to havemild or moderate disease and recover within one to two weeks of disease onset, severe cases may also be seen, especially who are with underlying conditions. Children with certain serious underlying conditions and who are <1 year of age are at higher risk for severe disease [3].6 Among 345 children from the United States with laboratory-confirmed COVID-19 and complete information about underlying conditions, 23% had an underlying condition. Common underlying conditions were chronic pulmonary disease (including asthma), cardiovascular disease, and immunosuppression (e.g., related to cancer, chemotherapy, radiation therapy, hematopoietic cell or solid organ transplant, high doses of glucocorticoids). Blood disorders, chronic kidney disease undergoing dialysis, chronic liver disease, pregnancy, endocrine disorders (e.g., diabetes mellitus), neurologic and neurodevelopmental conditions (e.g., cerebral palsy, epilepsy, intellectual disability, spinal cord injury) are significant reasons for severe disease.

Among the 295 pediatric cases for which information on both hospitalization status and underlying medical conditions was available, 28 of 37 (77%) hospitalized patients, (including patients admitted to an ICU), had one or more underlying medical condition; among 258 patients who were not hospitalized, 30 (12%) patients had underlying conditions. Three deaths were reported among the pediatric cases included in this analysis; however, the reason for death as COVID-19 is not confirmed yet.6 In the study by Lu et al., during hospitalization, 3 patients required intensive care support and invasive mechanical ventilation; all had coexisting conditions [hydronephrosis, leukemia (for which the patient was receiving maintenance chemotherapy), and intussusception] [30]. Epidemiologic and clinical features of pediatric case series are summarized in Table.

**Table 1:** Epidemiologic and clinical characteristics of pediatric patients.

		Dong et al.	Lu et al.	CDC/MMWR USA	Turkey*
Total confirmed patients		728 (confirmed) 1407 (suspected)	171	2572	117
Sex, male (%)		56.6	60.8	57	52.9
Median age (yr)		7 (IQR: 2–13)	6.7 (1d–15 yr)	11 (0–17)	8 (1d–17yr)
Age distribution (%)	<1 year 1-5 year 6-10 year 11-15 year >15 years	17.6 23 24.5 19.3 15.6	18.1 23.4 33.9 24.6 Not included	15 11 (1-4 yr) 15 (5-9 yr) 27 (10-14 yr) 32	13.6 20.5 16.2 28.2 21.3
Clinical presentation	Asymptomatic Mild disease Moderate disease Severe illness	4.4 51.0 38.7 7.4	15.8 19.3 - -	- - - -	- 50.4 - 0.8
Mortality (n)		1	1	3	0

## 6. Diagnosis

### 6.1. Diagnostic criteria

A diagnostic approach for COVID begins with the compatibility of exposure history, symptoms, and findings to the case definition criteria. Case definitions are based on the currently available data. Diagnostic criteria are regularly revised as new information accumulates. Definitions should be revised according to their local epidemiological data and individual factors. All countries are encouraged to publish definitions used online and in regular situation reports and to document periodic updates to definitions, which may affect the interpretation of surveillance data^9^9World Health Organization (2020). Global surveillance for COVID-19 caused by human infection with COVID-19 virus. Interim guidance 20 March 2020 [online]. Website https://www.who.int/docs/default-source/coronaviruse/global-surveillance-for-COVID-v-19-final200321-rev.pdf [accessed 12 April 2020]..

Pediatric patients should be evaluated according to complaints, clinical findings, and a history of exposure [35]. Firstly it should be stated if a child was in contact with a COVID-19 patientin the last two weeks period or has been to an endemic area for COVID-19. This information contributes to determine the level of risk, as low, medium, or high. Afterward, suspected cases are explored for the following: A- the presence of fever, any respiratory symptom, gastrointestinal symptoms like diarrhea. B- complete blood count should be tested to find out leukopenia, lymphopenia, and C-reactive protein is also tested in case of an increase. C- chest screening should be done to find out any infiltration if present. Further examination is carried out for suspected patients. If a case is positive for nCoV-2019 in nasal/pharyngeal swap or blood samples by polymerase chain reaction (PCR) assay OR if samples from the respiratory tract or blood samples are similar to nCoV-2019 is similar genetically, then the case is defined as confirmed [3].

Another critical issue in diagnosis is the method of optimal sampling choice for diagnosing COVID-19 [35]. In a study of 205 patients with COVID-19 who were sampled at various sites, the highest rates of positive viral RNA tests were reported from bronchoalveolar lavage (95 percent, 14 of 15 specimens) and sputum (72 percent, 72 of 104 samples). Still, oropharyngeal swap test results were as low as 32% [22]^10^10World Health Organization (2020). Report of the WHO-China joint mission on coronavirus disease 2019 (COVID-2019). February 16-24, 2020 [online]. Website http://www.who.int/docs/default-source/coronaviruse/who-china-jointmission-on-covid-19-finalreport.pdf [accessed 04. March 2020]..

### 6.2. Laboratory examinations

White blood cell count is normal in general. But leucopenia may be seen, with decreased lymphocyte count. C-reactive protein (CRP) may be normal or increased. Interleukin-6 (IL-6) is also generally high in patients, especially in severe cases. 

Procalcitonin (PCT) is normal in most cases, and PCT > 0.5 ng/mL might be a sign of secondary bacterial infection. Elevation of liver enzymes, muscle enzymes, and myoglobin, and increased level of D-dimer might be seen in severe cases [6].

### 6.3. Imaging features

In the early stage of pneumonia cases, chest images show multiple small patchy shadows and interstitial changes, remarkable in the lung periphery. Severe cases can further develop to bilateral multiple ground-glass opacity, infiltrating shadows, and pulmonary consolidation, with infrequent pleural effusion [30].

A Chest CT scan shows pathologic findings more clearly, including ground-glass opacity and segmental consolidation in bilateral lungs, especially in the lung periphery. In children with severe infection, multiple lobar lesions may be present in both lungs. In the Lu et al.’s study of pediatric cases, the most common radiologic finding was bilateral ground-glass opacity (32.7%). Other findings were local patchy shadowing 18.7%, bilateral patchy shadowing 12.3%, and interstitial abnormalities 1.2% [30]. Pleural effusion was not seen.

In a pediatric case series, among 15 confirmed pediatric COVID-19 cases, 6 patients had no lesions, while 9 patients had pulmonary inflammation lesions on their first chest CT images. Small nodular ground-glass opacities were found in 7 cases, and speckled ground-glass opacities were found in 2. Among the patients whose second PCR test was negative, chest CT images showed fewer lesions in 2 cases, no lesion in 3 cases, and no improvement in 1 case. The other 9 cases were still positive in the second nucleic acid test. Six of them showed similar chest CT inflammation, while 3 patients had new lesions, which were all small nodular ground-glass opacities [42]. 

## 7. Treatment

There are many published and ongoing studies about the treatment of COVID-19, but these studies are generally on adult patients. In the pediatric era, treatment strategies are commonly modified from adult reports, and future studies are warranted.

Home care facilities seem to be feasible for patients with asymptomatic infection, mild infection (e.g., fever, cough, and/or myalgias without dyspnea, hypoxia, and tachypnea), and having no underlying condition.These patients should adequately be isolated in the outpatient setting^11^11Centers for Disease Control and Prevention (2020). Interim clinical guidance for management of patients with confirmed coronavirus disease (COVID-19). Revisions were made on April 3, 2020 [online]. Website https://www.cdc.gov/coronavirus/2019-ncov/hcp/ clinical-guidance-management-patients.html [accessed 10 April 2020].,^12^12World Health Organization (2020).Home care for patients with COVID-19 presenting with mild symptoms and management of their contacts [online]. Website https://www.who.int/publications-detail/home-care-for-patients-with-suspected-novel-coronavirus-(ncov)-infection-presenting-with-mild-symptoms-and-management-of-contacts [accessed 10 April 2020]..

Treatment modalities may be summarized in the following subtitles. 

### 7.1. Symptomatic and supportive care

Symptomatic and supportive care is similar to other upper respiratory or gastrointestinal clinical syndromes like common cold or acute gastroenteritis. The general strategies include resting and supportive treatments, sufficient calorie, and water intake, maintaining water-electrolyte balance, and homeostasis [6]. Patients and household members should be educated about personal hygiene, infection control measures to prevent the infection from spreading to household contacts. Outpatients with COVID-19 should stay at home and try to separate themselves from other people and animals in the household. There is an extensive guideline prepared by WHO for home care patients.^12^ The optimal duration of home isolation is detailed in another section. 

### 7.2. Antibiotics

Antimicrobial stewardship is crucial. There is no need for antibiotic treatment routinely. Irrational use of antibiotics (especially broad-spectrum) should be avoided. Patients should be monitored carefully in case of bacterial coinfection. If there is clinical or laboratory evidence of secondary bacterial infection, appropriate antibiotics should be used appropriately [6]. 

### 7.3. Management for oxygen therapy and mechanical ventilatory support

Patients with respiratory distress and severe acute respiratory illness (SARI), including hypoxemia or shock, should be given supplemental oxygen therapy immediately, providing the level of >94%. Children with severe respiratory distress, central cyanosis, shock, coma, or convulsions should receive airway management and oxygen therapy. Nasal cannula is preferred in young children^13^13World Health Organization (2020).Clinical management of severe acute respiratory infection (SARI) when COVID-19 disease is suspected. Interim guidance 13 March 2020 [online]. Website https://www.who.int/publications-detail/clinical-management-of-severeacute- respiratory-infection-when-novel-coronavirus-(ncov)-infection-is-suspected [accessed 10 April 2020].. High-flow oxygen and noninvasive positive pressure ventilation have been used. But these procedures might cause aerosolization, and specific isolation precautions may be needed [43]. 

When respiratory distress keeps rising despite standard oxygen therapy, advanced oxygen/ventilatory support should be provided. If improvement is not possible, mechanical ventilation with endotracheal intubation and a protective lung ventilation strategy should be adopted [6].

### 7.4. Antiviral therapy

There is not an approved COVID-19 specific therapy for pediatric patients. Different agents were tried in some cases, but there is not sufficient data to make general recommendations for routine use. According to a recent report by Sanders, no proven effective therapies for this virus currently exist, and the most promising treatment was remdesivir [44]. Some of these agents will be discussed as follows.

#### 7.4.1. Hydroxychloroquine/chloroquine

There is not enough evidence showing whether hydroxychloroquine or chloroquine has a role in the treatment of COVID-19. There are in vitro studies that show both chloroquine and hydroxychloroquine inhibit SARS-CoV-2 in vitro, although hydroxychloroquine appears to be more potent [45]. Treatment guidelines from China’s National Health Commission supported the use of chloroquine. It was shown that the progression of disease was reduced, and the duration of symptoms decreased [46]. However, primary data supporting these claims have not been published yet. A randomized trial of patients with mild COVID-19 pneumonia without hypoxia reported that adding hydroxychloroquine to the standard of care resulted in clinical and laboratory improvement, but there are concerns about the methodology of this study^14^14Chen Z, Hu J, Zhang Z et al. Efficacy of hydroxychloroquine in patients with COVID-19: Results of a randomized trial. MedRxiv (2020). doi:10.1101/2020.03.22.20040758v2.

The Infectious Disease Society of America (IDSA) guideline panel for COVID-19 treatment, recommends hydroxychloroquine/chloroquine in the context of a clinical trial. But there is a knowledge gap^15^15Bhimraj A, Morgan RL, Shumaker AH, Lavergne V, Baden L et al. Infectious Diseases Society of America Guidelines on the Treatment and Management of Patients with COVID-19 - 2020 [online]. Website www.idsociety.org/COVID19guidelines [accessed 11 April 2020]..

There is not a specific dosage recommendation for hydroxychloroquine in pediatric patients. Some experts from Iran suggested to use, 3–5 mg/kg/day (max dose 400 mg) hydroxychloroquine sulfate iv, in pediatric patients, twice daily for five days with careful monitoring for cardiac arrhythmias including QT interval prolongation or torsades de pointes [47]. This drug may be used in pregnancy and lactation if the benefit outweighs risks [44]. 

Concurrent use of azithromycin with hydroxychloroquine for treating COVID-19 is not routinely recommended. One study suggested the use of azithromycin in combination with hydroxychloroquine was associated with more rapid resolution of virus detection than hydroxychloroquine alone [48]. Still, another small observational study in patients with more severe illness did not suggest rapid viral RNA clearance with the combination [49]. Both azithromycin and hydroxychloroquine are associated with QTc prolongation, and combined use may potentiate this adverse effect. The IDSA guideline panel recommends the use of the hydroxychloroquine and azithromycin combination only be used in the context of a clinical trial.^15^

#### 7.4.2. Interferon-α2b

According to Chen’s review in pediatric patients, interferon-α2b nebulization (100,000–200,000 IU/kg for mild cases, and 200,000–400,000 IU/kg for severe cases, two times/day for 5–7 days.) can be applied [6].

#### 7.4.3. Lopinavir-ritonavir 

Lopinavir ritonavir combination appears to have little to no role in the treatment of SARS-CoV-2 infection. This drug is a protease inhibitor and primarily used for HIV infection. According to a study, it has in vitro activity against the SARS-CoV [50] and appears to have some activity against MERS-CoV in animal studies [51]. Nevertheless, there was no significant improvement in clinical outcomes or mortality in a randomized trial of 199 patients between the lopinavir-ritonavir group and those who received standard of care alone [52].

Among patients who have been admitted to the hospital with COVID-19, the IDSA guideline panel recommends the combination of lopinavir/ritonavir only in the context of a clinical trial. There is also a knowledge gap in this condition.^15^

#### 7.4.4. Remdesivir 

Remdecivir is a novel nucleotide analog that has activity against SARS-CoV-2 in vitro and related coronaviruses (including SARS and MERS-CoV) both in vitro and in animal studies [53]. It is also potent in vitro activity against SARS-CoV-2, but it is not approved yet and currently, is being tested in ongoing randomized trials [44]. 

A recent case series of 53 patients with severe COVID-19 pneumonia who received remdesivir under a compassionate-use protocol reported clinical improvement in 68% after a median follow-up of 18 days, with 13% mortality and a generally acceptable toxicity profile [54]. However, there was no comparison group of similar patients who received standard care at the participating institutions.

Efficacy in pediatric patients and optimal dosing are not established yet. A dosing regimen of 5 mg/kg/dose iv once daily (max: 200 mg) on day 1, followed by 2.5 mg/kg/dose iv once daily (max: 100 mg) was used in 41 pediatric patients (including 2 neonates) who received remdesivir in a phase 3 Ebola study^16^16National Institute of Allergy and Infectious Diseases (NIAID) (2020). Investigational Therapeutics for the Treatment of People With Ebola Virus Disease. [online]. Website https://clinicaltrials.gov/ct2/show/NCT03719586? term=randomized+ebola draw=2 [accessed 30 March 2020]..The optimal duration of therapy for COVID-19 is unknown.

#### 7.4.5. Favipiravir 

Favipiravir was approved for the treatment of novel influenza in China. Clinical trials of favipiravir for treatment of COVID-19 are going on, but there is not any evidence for pediatric patients yet. It is an RNA polymerase inhibitor. It is thought to have potential antiviral action on SARS-CoV-2. In February, a clinical trial on favipiravir for the treatment of COVID-19 was initiated, and results were promising. The preliminary results from a total of 80 patientsindicated that favipiravir had more potent antiviral action than lopinavir/ritonavir^17^17News (in Chinese) [online]. Website http://www.szdsyy.com/News/0a6c1e58-e3d0-4cd1- 867a-d5524bc59cd6.html [accessed 22 March 2020]. . There is no specific dosing recommendation for favipiravir in pediatric COVID-19 patients, but doses that were used in the treatment of the Ebola virus or influenza virus may be appropriate.

### 7.5. Glucocorticoids

WHO and CDC claim that systemic glucocorticoids should not be used in patients with COVID-19, unless there are other indications (e.g., exacerbation of chronic obstructive pulmonary disease). A pediatric review reports that they can be used in severe cases when indicated, but its efficacy needs further evaluation [6]. Many studies on SARS and MERS-CoV showed a lack of effectiveness and possible harm [55]. The IDSA guideline panel also suggests against the use of corticosteroids (conditional recommendation, very low certainty of the evidence). The same guideline recommends the use of corticosteroids for patients only with ARDS due to COVID-19 in the context of a clinical trial.^15^

### 7.6. Tocilizumab

Tocilizumab is an IL-6 receptor inhibitor used for rheumatic diseases and cytokine release syndrome. It was reported that severe COVID-19 patients had elevated IL-6 levels, and there are case reports which describe good outcomes with tocilizumab [56]. Tocilizumab may be recommended for severe COVID-19 patients and patients with high IL6 levels^18^18Reuters (2020). China approves use of Roche drug in battle against coronavirus complications [online]. Website https://www.reuters.com/article/us-health-coronavirus-china-roche-hldg/china-approves-use-of-roche-arthritis-drug-for-coronavirus-patientsidUSKBN20R0LF [accessed 10 April 2020].. The IDSA guideline panel recommends tocilizumab only in the context of a clinical trial. Xu et al. reported that treatment with tocilizumab reduced mortality^19^19Xu X, Han M, Li T, Sun W, Wang D et al. Effective treatment of severe COVID-19 patients with tocilizumab. ChinaXiv 2020; 202003.00026v1. doi: 10.1101/2020.04.08.029769. However, this conclusion remains highly uncertain, given the lack of a contemporaneous control or adjustments for confounding factors.

### 7.7. Organ function support

WHO recommends recognizing septic shock in children with any hypotension or two or more of the following: altered mental state; tachycardia or bradycardia; prolonged capillary refill (> 2 s) or feeble pulses; tachypnea; mottled or cold skin or petechial or purpuric rash; increased lactate; oliguria; hyperthermia or hypothermia.^13^ Antimicrobial therapy should be started, and fluid bolus should be initiated. In case of circulation dysfunction, vasoactive drugs may be used to improve microcirculation [6]. In children, epinephrine is considered first-line, while norepinephrine can be added if shock persists despite the optimal dose of epinephrine. Continuous blood purification should be considered in cases of multiple organ failure (especially acute kidney injury), or capacity overload and life-threatening imbalance of water, electrolyte, and acid-base. If combined with liver failure, plasma exchange is feasible.^13^ Extracorporeal membrane oxygenation (ECMO) has been proven to be an effective therapy in the treatment of respiratory failure ARDS, so it may also be effective in the treatment of severe COVID-19. Since there is a lack of clinical trial of ECMO on COVID-19, we could not conclude whether SARS-CoV-2-infected patients have benefited from ECMO at this time [57].

### 7.8. Intravenous immunoglobulin and Immun Convalescent Plasma [58]

In this current pandemic, there are reports that convalescent plasma has been used in China to treat patients with COVID-19 [59]. 

In a pilot study of 10 patients with severe COVID-19, the investigators collected convalescent plasma with neutralizing antibody titers at or exceeding a 1:640 dilution^20^20Duan K, Liu B, Li C, Zhang H, Yu T et al. The feasibility of convalescent plasma therapy in severe COVID-19 patients: a pilot study. MedRxiv 2020. doi: 10.1101/2020.03.16.20036145. Transfusion of convalescent plasma resulted in no serious adverse effect in the recipients. Improvement was shown in symptoms within 1–3 days of transfusion; they also demonstrated radiological improvement in pulmonary lesions. Although there are methodologic limitations, findings suggest that the administration of convalescent plasma is safe, reduces viral load, and may improve clinical outcomes. Similarly, high-dose intravenous immunoglobulin (IVIg) has been suggested as a potential therapy for COVID-19 [60]; however, supporting data are few and marred by potential confounders.

## 8. Conclusion

As the whole world is going through hard times because of the pandemic, health care workers are carrying out a considerable effort to find new treatment modalities. There is a very dynamic process on epidemiology and clinical trials about the current pandemic globally. Many reports are coming out every day. All these efforts are for saving more lives. According to the data presented in this review, this pandemic seems to have less risk for children than adults. The main point for COVID-19 infection is ‘not to be infected.’ Infection control precautions should be carried out carefully. Herein, also, treatment recommendations are summarized. In severe cases, antiviral drugs may also be used, but it should be kept in mind that none of these are proved for pediatric patients yet and not approved. We wish ease and success to all healthcare workers, governments, and humanity in fighting this pandemic.

## Acknowledgments

Hasan TEZER is a member of the Advisory Committee of the Ministry of Health of Turkey.
